# Supplementary material for: Effects of Trichoderma harzianum combined with Phanerochaete chrysosporium on lignin degradation and humification during chicken manure and rice husk composting
Source: Front Microbiol. 2025 Feb 28;16:1515931. doi: 10.3389/fmicb.2025.1515931 (PMC11906335; doi:10.3389/fmicb.2025.1515931)
Supplement: Supplementary file 1 [file Data_Sheet_1.zip › 6.Function/CKF_HF/FunGuild/l2/metastats/Litter_Saprotroph.HF_CKF.metastats.pdf]

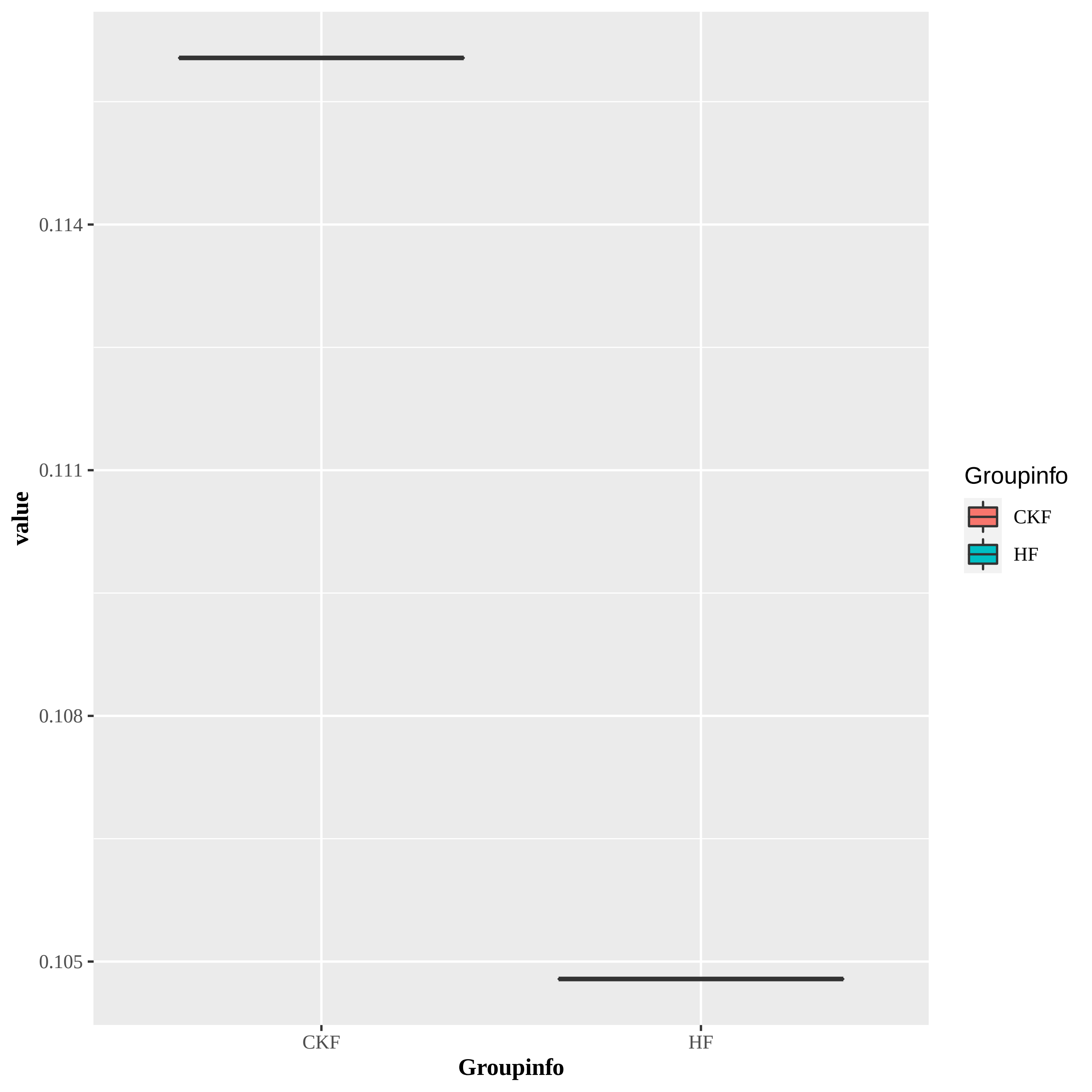

0.114
0.111
0.108
0.105
value
CKF
HF
Groupinfo
Groupinfo
CKF
HF
